# Vascular Calcification: Molecular Networking, Pathological Implications and Translational Opportunities

**DOI:** 10.3390/biom14030275

**Published:** 2024-02-25

**Authors:** Miguel A. Ortega, Diego De Leon-Oliva, Maria José Gimeno-Longas, Diego Liviu Boaru, Oscar Fraile-Martinez, Cielo García-Montero, Amador Velazquez de Castro, Silvestra Barrena-Blázquez, Laura López-González, Silvia Amor, Natalio García-Honduvilla, Julia Buján, Luis G. Guijarro, Elisa Castillo-Ruiz, Miguel Ángel Álvarez-Mon, Agustin Albillos, Melchor Álvarez-Mon, Raul Diaz, Miguel A. Saez

**Affiliations:** 1 CIBEREHD, Department of Medicine and Medical Specialties, Faculty of Medicine and Health Sciences, University of Alcalá, 28801 Alcala de Henares, Spain; diegodleonoliva01@gmail.com (D.D.L.-O.); diego.boaru@edu.uah.es (D.L.B.); oscarfra.7@hotmail.com (O.F.-M.); cielo.gmontero@gmail.com (C.G.-M.); avelazquezcb@gmail.com (A.V.d.C.); silviamortejero@hotmail.com (S.A.); natalio.garcia@uah.es (N.G.-H.); mjulia.bujan@uah.es (J.B.); elisa.castillo@uah.es (E.C.-R.); maalvarezdemon@icloud.com (M.Á.Á.-M.); agustin.albillos@uah.es (A.A.); mademons@gmail.com (M.Á.-M.); msaega1@oc.mde.es (M.A.S.); 2 Ramón y Cajal Institute of Sanitary Research (IRYCIS), 28034 Madrid, Spain; silvebarrena@gmail.com (S.B.-B.); laura.lgonzalez@uah.es (L.L.-G.); luis.gonzalez@uah.es (L.G.G.); raul.diazp@uah.es (R.D.); 3 Department of Cell Biology, School of Medicine, Universidad Complutense de Madrid, 28040 Madrid, Spain; margim08@ucm.es; 4 Department of Nursing and Physiotherapy, Faculty of Medicine and Health Sciences, University of Alcalá, 28801 Alcala de Henares, Spain; 5 Department of Surgery, Medical and Social Sciences, Faculty of Medicine and Health Sciences, University of Alcalá, 28801 Alcala de Henares, Spain; 6 Unit of Biochemistry and Molecular Biology, Department of System Biology (CIBEREHD), University of Alcalá, 28801 Alcala de Henares, Spain; 7 Immune System Diseases-Rheumatology Service, University Hospital Principe de Asturias, 28801 Alcala de Henares, Spain; 8 Surgery Service, University Hospital Principe de Asturias, 28801 Alcala de Henares, Spain; 9 Pathological Anatomy Service, Central University Hospital of Defence-UAH Madrid, 28801 Alcala de Henares, Spain

**Keywords:** vascular calcification, signaling pathways, endothelial cells (ECs), vascular smooth muscle cells (VSMCs), biomarkers

## Abstract

Calcification is a process of accumulation of calcium in tissues and deposition of calcium salts by the crystallization of PO_4_^3−^ and ionized calcium (Ca^2+^). It is a crucial process in the development of bones and teeth. However, pathological calcification can occur in almost any soft tissue of the organism. The better studied is vascular calcification, where calcium salts can accumulate in the intima or medial layer or in aortic valves, and it is associated with higher mortality and cardiovascular events, including myocardial infarction, stroke, aortic and peripheral artery disease (PAD), and diabetes or chronic kidney disease (CKD), among others. The process involves an intricate interplay of different cellular components, endothelial cells (ECs), vascular smooth muscle cells (VSMCs), fibroblasts, and pericytes, concurrent with the activation of several signaling pathways, calcium, Wnt, BMP/Smad, and Notch, and the regulation by different molecular mediators, growth factors (GFs), osteogenic factors and matrix vesicles (MVs). In the present review, we aim to explore the cellular players, molecular pathways, biomarkers, and clinical treatment strategies associated with vascular calcification to provide a current and comprehensive overview of the topic.

## 1. Introduction to Pathological Calcification

Calcification is a process of accumulation of calcium in tissues and deposition of calcium salts by the crystallization of PO_4_^3−^ and ionized calcium (Ca^2+^). While it serves as an essential mechanism for bone and teeth development and maturation, pathological calcification can manifest in almost all soft tissues, which is associated with aging and a variety of diseases [1]. In bone physiology, calcification plays a pivotal role in skeletal growth and strength [2]. This process involves the deposition of calcium phosphate crystals within a matrix, transforming into a rigid and resistant skeletal framework, and it is finely regulated by a network of osteoblasts, osteoclasts, stem cells, and signaling molecules, ensuring the formation of robust bone tissue [3]. Proteins and inorganic crystals combine to form the composite tissues of mammalian teeth and bones. These tissues are made up of about 70% inorganic materials, 20% proteins, and 10% water by weight [4]. Inorganic crystals resist compressive stresses and increase tissue toughness by depositing on matrix proteins; type I collagen is the main protein component of the matrix, and hydroxyapatite is the main inorganic component of the tissues [5].

Aging is the primary cause of pathological calcification, although tumors, blood vessels, and joints are often associated with this process [6]. Pathological calcification occurs through various pathways with varying levels of cellular control in non-skeletal tissues such as the vasculature and neoplasms [7]. Calcified deposits can be adverse by causing mechanical stress or stiffness in affected tissues and have been linked to cellular damage and inflammation, although they are often used as a marker of disease [8]. Calcified deposits are calcium phosphate crystals anchored within the extracellular matrix.

**Figure 1:**
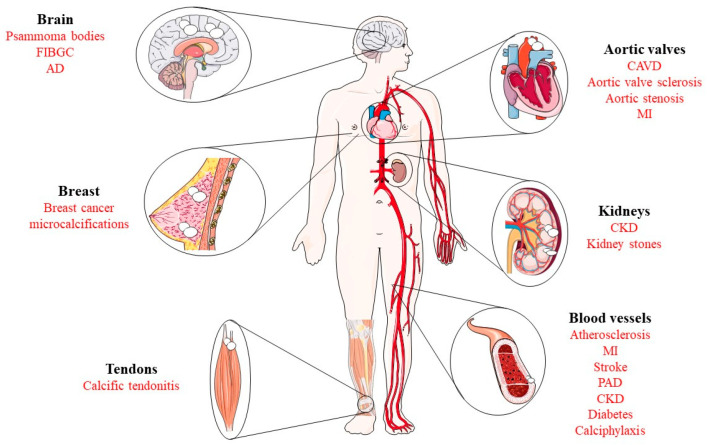
Schematic representation of the main anatomical locations susceptible to the deposition of calcium salts: blood vessels, aortic valves, breast cancer, brain, tendons, or kidneys. In black appear the anatomical locations of calcium deposits and in red the related diseases. FIBGC: familial idiopathic basal ganglia calcification, AD: Alzheimer’s disease, CAVD: calcific aortic valve disease, MI: myocardial infarction, CKD: chronic kidney disease, PAD: peripheral artery disease.

Depending on the conditions leading to the development of calcium phosphate crystals, dystrophic calcification manifests as the accumulation of calcium in regions affected by trauma or necrosis, which is derived from various causes such as blunt trauma, inflammation, injections, and the presence of parasites, with normal plasma levels of calcium and phosphate [9]. In contrast, metastatic calcification occurs when abnormalities in the levels of calcium and phosphate ions in the blood serum, i.e., hypercalcemia, lead to calcification in previously normal tissue [10,11,12]. Furthermore, iatrogenic calcification can be caused by some medical procedures, such as surgery, radiation therapy, or the administration of calcium or phosphate-containing agents [13]. For example, the calcification of skin known as calcinosis cutis appears after patients with tuberculosis receive intravenous calcium gluconate, calcium chloride, and para-aminosalicylic acid [14]. Although pathological calcification can occur in almost all soft tissues of the body, the most prone areas are blood vessels, heart valves, brain, breast, kidneys, gastric mucosa, lungs, and tendons, and research has focused primarily on vascular calcification [15,16,17,18,19,20,21,22] (Figure 1).

The mineral produced in ectopic calcification is less ordered, has variable crystal sizes and shapes, and is composed of calcium phosphate crystals and other calcium salts in contrast to physiological mineralization [23]. Glycosaminoglycans are present on the surface of hydroxyapatite crystals in both blood vessel calcification and bone mineralization, according to chemical and nuclear magnetic resonance (NMR) investigations. In general, calcium is deposited in the form of fine white granules or gritty clumps [24]. Microscopically, in sections stained with hematoxylin and eosin, calcium appears as basophilic, amorphous granular, or agglomerated and appears in black with the von Kossa stain. Interestingly, the presence of ribosomal RNA (rRNA) derived from ribosome degradation and nuclear chromatin contributes to aortic valve calcification through the stimulation of hydroxyapatite nucleation capacity exerted by the cell membrane-derived substance containing acidic phospholipid substrates (PPM/PPLs) with their anionic charges [25].

The deposition of calcium salts in blood vessels is known as vascular calcification. The calcium phosphate deposits can be found in the medial and intimal layers and aortic valves [26,27]. The intimal calcification is associated with atherosclerosis. Normally, the calcium salts are formed in the ECM rather than intracellular in the intimal layer, which is associated with atherosclerotic plaques, medial layer, or aortic valves [28]. The mineral phases can be apatite, whitlockite, or octacalcium phosphate ranging in sizes from submicron to larger than 0.5 mm [29]. Vascular calcification together with remodeling of the extracellular matrix (ECM) results in arterial stiffness and contributes to vessel rupture [30]. Recent findings with Raman spectroscopy indicate an interaction between intimal and medial calcifications related to atherosclerosis, demonstrating an elevation of the apatite/whitlockite ratio in the aortic media precisely beneath an atherosclerotic plaque [31]. Medial calcification, also termed Mönckeberg sclerosis, occurs preferentially along the elastic lamina and is usually identified in small and medium-sized arteries of the lower extremities. It is associated with advanced age, diabetes, and chronic kidney disease (CKD), and, unlike intimal calcification, it occurs independently of atherosclerosis [32,33,34]. It is a process similar to intramembranous bone formation, involving the transdifferentiation of vascular smooth muscle cells (VSMCs) to mineralizing cells. Arterial stiffness and calcification of the arterial media create a vicious circle, in which ECs are often the initiators [35]. The calcific aortic valve disease (CAVD) is the cause of the thickening, fibrosis, and mineralization of the aortic valve leaflets, which ranges from aortic sclerosis (mild valve thickening) to aortic stenosis (severe calcification with impaired leaflet motion) [36,37,38,39]. The valve endothelial and interstitial cells and immune cells promote the remodeling of aortic valve leaflets. Atherosclerotic risk factors contribute to the risk of CAVD, such as age, smoking, hypertension, hyperlipidemia, and diabetes [40].

On the other hand, the structure–function relationship of soft tissue calcification is a very dynamic and intricate process that still requires further understanding. Different cellular types play a role in the process and multiple signaling pathways have been recognized, including Ca^2+^ signaling, Wnt/β-catenin, BMP/Smad, and Notch pathways, and different molecular mediators are involved, such as osteogenic factors, growth factors (GFs) or matrix vesicles (MVs) [1,41,42,43,44,45]. The study of the molecular pathways may drive the exploration of potential therapeutic targets and the use of proper biomarkers. Therefore, the present review aims to explore the cellular players, molecular pathways, biomarkers, and clinical treatment strategies associated with calcification to provide a current and comprehensive overview of the topic.

## 2. Cellular Players in Vascular Calcification

Several actors are involved in soft tissue calcification: not only cells but also cell matrix components. In this section, we will center on the main cell types playing a role in the deposition of calcium, including specialized cells in which changing microenvironments led to an osteogenic-like phenotype. The study of the mechanisms governing vascular calcification is difficult, which is mainly due to the unresolved origin of calcified cells within cardiovascular lesions. These cells could originate from various sources: VSMCs undergoing dedifferentiation and proliferation, bone marrow-derived stem cells present from the bloodstream, or multipotent calcifying vascular cells resident in the vascular wall which can differentiate into osteoblasts and chondrocytes with the contribution of endothelial cells, macrophages, pericytes, and fibroblasts [46]. These cells are characterized by the expression of calcification markers, including some transcription factors such as Msx2 (Msh homeobox 2) and Runx2, which are both responsible for the osteogenic phenotype, Cbfa1 (core-binding factor α1), SP7/Osterix or SOX9, some proteins such as osteopontin (OPN), osteocalcin (OC), alkaline phosphatase (AP), bone sialoprotein and collagens II and X, while the contractile markers are decreased or lost [47,48]. Runx2 is the principal activator of mineralization, as it is the transcription factor that upregulates the expression of osteoblastic differentiation genes, e.g., osteocalcin and osteopontin.

### 2.1. Endothelial Cells

ECs are the vascular inner lining epithelium and have a critical role in the maintenance of blood flow. The EC plasticity is responsible for vascular calcification, specifically the endothelial-to-mesenchymal transition, which is a phenomenon also found in normal biological processes such as embryonic development. In both cases, ECs lose their cell features and acquire new markers that characterize the osteogenic lineage through the increase in the osteocalcin expression in areas where stretching occurs [49,50]. These places will be areas for ectopic calcification due to the EC dysfunction that affects endothelial secretory function and inflammatory regulation [51]. The human umbilical vein ECs (HUVECs) could suffer a mesenchymal transition through the overexpression of OCT-4 in the presence of some members of the transforming growth factor-β (TGF-β) family, specifically bone morphogenetic protein type 4 (BMP4), to induce osteogenesis in vitro experiments [52].

### 2.2. Vascular Smooth Muscle Cells

Vascular smooth muscle cells (VSMCs) form the middle layer of the vascular tree or the contractile layer of the digestive or genitourinary systems, contributing to the optimal functioning of the body. In the vascular system, the VSMCs are the major cellular component of the middle layer and are directly involved in the intimal metastatic calcification in several pathologies, because these cells can switch toward an osteogenic phenotype when the deposition of calcium increases in these tissues [53,54]. Sometimes, VSMCs can become foam cells changing the expression of SMCs markers as α-smooth actin and increasing macrophage markers as CD68+ and ABCA1 [55]. In addition, interaction between ECs and VSMCs can exacerbate arterial calcification in cases of hyperphosphatemia [56]. This occurs through the release of endothelial cell-derived exosomal miR-670-3p, which targets IGF-1 upon uptake by VSMCs. The osteoblastic/chondrogenic differentiation of VSMCs is regulated by various factors, including mechanical, biochemical, and molecular signals. Mechanical forces, such as transmural pressure, pulsatile pressure, and shear stress, promote dedifferentiation toward the synthetic phenotype through the mechanotransduction signals from the cytoskeleton [53,57,58]. Hypoxia within the vascular microenvironment can trigger signaling cascades that contribute to the differentiation of VSMCs toward a chondrogenic phenotype [59]. The mechanisms underlying VSMC calcification under hypoxic conditions require either hypoxia-inducible factor (HIF) activation or the production of mitochondrial-derived reactive oxygen species (ROS) [60,61]. High levels of inorganic phosphate (Pi) increase VSMC migration and calcification and are related to impaired Pi/calcium balance and miR-223 involvement, whereas TGF-β/Smad3 signaling plays an inhibitory role in Pi-induced VSMC calcification [62,63,64]. Various cytokines and GFs regulate VSMC differentiation, such as TNF-α, IL-1β, IL-6 or TGF-β. The tumor necrosis factor-α (TNF-α) and transforming growth factor-β1 (TGF-β1) enhance the VSMC calcification through their receptors [54,65,66]. M1 macrophages secrete TNF-α, and it is upregulated by miR32-5p in the microenvironment [67,68].

### 2.3. Macrophages

Macrophages are antigen-presenting cells (APCs) involved in inflammation processes releasing different cytokines; these cells detect phagocyte-damaged cells and pathogens, such as bacteria. Several subsets of macrophages are observed in tissues, depending on the lesion characteristics, being M1 and M2 as the predominant subtypes. M1 macrophages are considered classically activated and exhibit a proinflammatory profile, being responsible for removing pathogens and the release of ROS, and they are associated with the presence of microcalcifications; M2 macrophages or activated macrophages have an anti-inflammatory profile and promote tissue repair, being involved in the macrocalcification development [69,70]. Their plasticity is a key in some pathological processes to control the progression of abnormalities in tissues, as in the atherosclerotic plaque, where M1 is predominant in the lesion shoulder, M1 and M2 in the necrotic core, and M2 in the adventitia [71]. In addition, the calcium–phosphate imbalance impairs the phagocytic clearance of VSMCs-derived apoptotic bodies (ABs) by macrophages and promotes the release of pro-calcific MVs [72,73]. The accumulation of ABs serves as a nucleation site for the deposition of Ca/P nanocrystals, contributing to vascular calcification [74,75].

### 2.4. Pericytes and Fibroblasts

Pericytes are perivascular cells covering microvascular capillaries, and they are considered as a reservoir of precursor cells. These cells have been shown to have an osteogenic potential in a model of in vivo osteogenesis, forming mineralized nodules, and they have been able to express osteoblastic markers such as osteonectin, OPN, osteocalcin, and bone sialoprotein [76,77]. In addition, pericytes by the secretion of osteoprotegerin (OPG) may be involved in the formation of osteoid metaplasia [78].

Fibroblasts can acquire a “myofibroblast” phenotype, essentially muscle-fibroblast intermediate cells with contractile properties, which are connected to vascular calcification. These present the capacity to transition into calcifying osteoblast-like cells under specific conditions and mechanical and inflammatory stresses [79]. In these processes, TNF-α, elastin degradation products, TGF-β1 or ROS are involved [80,81].

## 3. Molecular Networking in Vascular Calcification

### 3.1. Signaling Pathways in Vascular Calcification

Different signaling pathways and molecular mediators lead to both physiological and pathological tissue calcification. Primarily, the activation of fundamental pathways such as calcium, Wnt/β-catenin, BMP/Smad, and Notch initiate this phenomenon [1,42,82,83]. In addition, molecular mediators regulate this process, including osteogenic factors, GFs, and MVs [84,85,86].

The calcium ion is the most versatile cellular messenger, and its functions are cellular communication, intracellular signaling, neuronal function, muscle contraction, cell cycle regulation, cell death, or enzyme activation [87,88]. Extracellular calcium serves as the primary initiator of vascular calcification. Dysfunctions in calcium regulatory proteins, including calcium-sensing receptors (CaSRs), calmodulin, and other associated molecules, lead to pathological calcification. It has been observed an increased CaSR expression in valvular interstitial cells (VICs) of calcified aortic valves and its modulation of CaSR expression in these cells has been shown to reduce calcium-induced valve calcification in vitro [89]. The upregulation of CaSR expression in VSMCs reduces cellular calcium deposition and difficulties in their transition to a calcifying phenotype [90]. VEGF-driven Runx2, a marker of calcification whose expression in VICs operates through the IP3R/CaMKII/CREB axis and, in hypoxic conditions, miR-7-5p, facilitates osteoblastic differentiation and the calcification of human aortic smooth muscle cells (HASMCs) by activating calponin-3 and CaMKII [91,92]. Also, the calcium-dependent cytosolic phospholipase A2α (cPLA2α) contributes to aortic valve calcification in vitro [93].

The Wnt pathway plays diverse functions in embryonic development and adult homeostasis [94]. The activation of different Wnt signaling pathways promotes vascular calcification and valve sclerosis through different mechanisms [95]. The canonical Wnt/β-catenin functions by the binding of an extracellular Wnt ligand to a seven-pass transmembrane Frizzled (Fz) receptor and co-receptor low-density lipoprotein receptor-related protein (LRP5/6) activating β-catenin. Inside the nucleus, β-catenin forms a transcriptional complex with LEF-1/TCF DNA-binding transcription factors and induces the expression of specific target genes. In the case of pathological calcification, it upregulates markers of calcification, such as Runx2, BMPs, PPAR, or OPN [82,95]. On the other hand, the non-canonical Wnt signaling pathway stimulates pathological calcification through the Ror1/2 co-receptor and activates both branches, the Planar Cell Polarity pathway or PCP pathway and the Wnt/Ca^2+^ pathway [96,97]. The former triggers the activation of small GTPases Rho and Rac, leading to alterations in the cell cytoskeleton and lateral asymmetry. The latter implies the increase in cytosolic Ca^2+^ through phospholipase C (PLC), which activates both calmodulin-dependent protein kinase II (CamKII) and protein kinase C (PKC) [98]. These lead to the upregulation of target genes via the activation of transcription factors, such as nuclear factor-κB (NF-κB) or cAMP-responsive element binding.

Bone morphogenetic proteins (BMPs) are members of the TGF-β superfamily essential for development, but they also participate in the regulation of cardiovascular structure and function. The extracellular binding of BMP to type I and type II receptors leads to the formation of heteromeric signaling complexes [99,100]. The canonical pathway induces the phosphorylation of Smad1/5/8 (R-Smad), which binds to Smad4 and the complex translocates into the nucleus to regulate gene expression [101]. The Smad-independent non-canonical pathway activates the ERK, JNK, and p38 MAPK signaling pathways, leading to the phosphorylation of several transcription factors (TF), such as serum response factor (SRF), ternary complex factor (TCF) family members, activator protein 1 (AP1) complexes and activating transcription factor 2 (ATF2) [102,103]. Several investigations have highlighted the ability of BMP-2, BMP-4, and BMP-6 to stimulate the development of vascular calcification in association with atherosclerosis [104,105]. These BMP ligands direct osteogenic programming and promote the expression of Runx2 and Msx2. The receptor activator of nuclear factor κB ligand (RANKL) increases VSMC calcification by promoting BMP-4 expression [106]. Altered blood flow in large vessels downregulates KLF2 expression, leading to endothelial–mesenchymal transition (EndoMT) and increased vascular calcification through BMP-4/Smad1/5 signaling [107]. BMP-6 and ox-LDL synergistically induce osteogenic differentiation and mineralization, highlighting an important connection between BMP signaling, oxidative stress, and inflammation in vascular calcification associated with atherosclerosis [108]. Conversely, BMP-7 exogenous administration has demonstrated anti-calcifying effects in rodent models of uremia, where it attenuates vascular calcification together with the downregulation of Runx2 and osteocalcin [109,110].

Lastly, Notch signaling is crucial for development, differentiation, and tissue homeostasis. It consists of a juxtacrine signaling between Notch transmembrane receptors making contact with Notch transmembrane ligands in neighboring cells. This interaction triggers the proteolytic cleavage of the Notch receptor, resulting in the release of the Notch intracellular domain (NICD). This acts as a transcription regulator of CSL, HES/Hey, and Runx2 [111,112]. The Notch/RBP-Jk signaling directly upregulates *Msx2* gene expression in VSMCs [83]. On the other hand, Notch presents inhibitory effects of vascular calcification. The activation of Notch1 induced by USP9X S-nitrosylation inhibits the calcification of porcine aortic VIC regulated by nitric oxide (NO) [113]. Elevated Wnt16 levels stimulate Notch activity and counteract TGFβ3-induced Notch suppression in VSMCs [114]. This process leads to reduced chondrogenesis in VSMCs exposed to TGFβ3, suggesting that the absence of Wnt16 plays a pivotal role in TGFβ3-induced chondrogenic change in VSMCs. Moreover, exosomal Notch3 released by endothelial cells, when present in high glucose environments, decreases vascular calcification by inhibiting mTOR [115]. Lastly, the induction of Notch1 and matrix γ-carboxyglutamate (Gla) protein (MGP) by shearing results in the downregulation of osteoblast-like genes in human aortic valve endothelial cells [116].

### 3.2. Molecular Mediators of Vascular Calcification

In addition to the above-mentioned signaling pathways, multiple molecular mediators play an important role in the process of vascular calcification, including GFs, osteogenic factors, and MVs. Within the GFs, some of the ones that stand out are TGF-β, in particular the aforementioned BMPs, platelet-derived growth factor (PDGF), fibroblast growth factor 21 and 23 (FGF21/23), and vascular endothelial growth factor (VEGF). PDGF accelerates vascular calcification through different mechanisms that combine the induction of inflammation (IL-1β, IL-6, MCP-1, and ICAM-1), oxidative stress (ROS), phenotype transition (BMP2, Pit-1, OPG, and CNP), and mesenchymal stem cells (MSCs) migration A [117]. Elevated levels of circulating FGF23 and its overexpression in blood vessels are associated with vascular calcification [85]. Contrary to this, FGF21 has gained special attention due to its anti-calcifying effects [118,119]. In rats, it alleviates the endoplasmic reticulum stress-mediated apoptosis pathways. Lastly, it has been proposed that VEGF induces Ca^2+^/CaMKII activation through VEGF receptor 2, leading to the upregulation of Runx2 and therefore vascular calcification [92].

The osteogenic factors are those that regulate the process of calcification, both promoters and inhibitors. Among these promoters, cadherin-11 (Cad11) is a cell–cell adhesion protein related to inflammation in rheumatoid arthritis. Cad11 induces aortic valve calcification through Rac1, and its overexpression leads to the upregulation of RhoA and Sox9 and extracellular matrix remodeling [120,121]. Calcific nodule morphogenesis by valvular myofibroblasts requires robust cell–cell connections regulated by Cad11 [122,123]. In contrast, the several osteogenic factors that inhibit vascular calcification include MGP, OPN, OPG, fetuin-A, and also small molecules, such as inorganic pyrophosphate (Ppi), bisphosphonates, and magnesium [124,125]. MGP has been proposed to act through the direct inhibition of calcium–phosphate precipitation, the formation of MVs, the formation of ABs, and the transdifferentiation of VSMCs, and it is an independent predictor of both intimal and medial vascular calcification in CKD [126,127,128]. Phosphorylated OPN acts as a physiological inhibitor of vascular calcification and reduces inflammation factors in osteoclast formation by the regulation of macrophage activation in hypertensive patients [129,130]. Endogenous arterial OPG shows a protective role against vascular calcification through different mechanisms in animal models, such as the inhibition of ALP-mediated osteogenic differentiation of vascular cells and downregulation of the Notch1–RBP–Jκ signaling pathway, while in humans, plasma OPG is probably produced as a response against vascular calcification [131,132,133].

MVs are tiny extracellular structures originating from chondrocytes and osteoblasts, essential in early mineralization, especially in the formation of hydroxyapatite. Ranging in size from 100 to 700 nm, MVs contain hydroxyapatite nanocrystals, which are observed in aortic and media valve calcification and atherosclerotic lesions [45]. Unlike spherical particles in aortic valve calcification, MVs are observed in vessel walls with a hollow core and amorphous minerals [86]. Their formation is linked to intercellular calcium signaling and contains calcium-binding annexins and alkaline phosphatase, which hinders the action of pyrophosphate and favors hydroxyapatite formation [134]. VSMCs regulate mineralization; under healthy conditions, inhibitors prevent their calcification [135]. VSMCs in atherosclerosis and CKD lack mineralization despite their presence in the tissue, which is associated with elastin and collagen fibrils [136]. Elevated extracellular calcium triggers VSMC responses, influencing MV-related calcification, but the exact mechanisms are under exploration.

Also, MVs released locally or the presence of circulating nucleation complexes serve as sites for calcium complex crystallization. Indeed, increased bone turnover, such as in the case of postmenopausal osteoporosis, is related to the release of circulating nucleation complexes that contribute to vascular calcification [137,138]. The process of bone remodeling regulates the calcium levels in the body and is masterly modulated by the OPG–RANK–RANKL system [139].

The vascular calcification process is influenced by many more factors, which present a balance between inhibitors and promoters (some of them are reviewed below). This is impaired under pathological conditions, leading to a decrease in protective inhibitors. Among the factors known to decrease calcification are osteopontin, OPG, BMP-7, magnesium ions (Mg^2+^), osteonectin, vitamin K, high-density lipoprotein cholesterol (HDL-C), growth arrest-specific protein 6, albumin, parathyroid hormone, parathyroid hormone-related peptide, phosphonoformic acid, C-type natriuretic peptide, and adrenomedullin [130,133,140,141,142,143,144,145,146,147,148,149,150,151].

Conversely, factors that promote calcification include TNF-α, TGF-β, ROS, platelet-derived growth factor (PDGF), cadherin-11 (cad-11), the BMP-2/Smad pathway, klotho, IL-1, -4, and -6, oxidized and acetylated low-density lipoprotein cholesterol (LDL-C), C-reactive protein (CRP), leptin, advanced glycation end products, glucocorticoids, type I collagen, fibronectin, 25-hydroxycholesterol, 17β-estradiol, uremic serum, 1,25-dihydroxycholecalciferol, and cyclic adenosine monophosphate (cAMP) [117,120,152,153,154,155,156,157,158,159,160,161,162,163,164,165,166,167,168,169].

## 4. Pathological Implications

Pathological calcification does not usually cause clinical dysfunction, but significant deposits in organs can cause organ damage, such as nephrocalcinosis, leading to renal failure. Calcium deposition decreases the mechanical elastance of the arteries required for physiological functions; this is especially risky for the aorta, which has an impact on cardiovascular hemodynamics and contributes to significant morbidity and mortality [26]. Vascular calcification has been related to different cardiovascular diseases, including myocardial infarction (MI), stroke, aortic and peripheral arterial disease, heart failure, chronic kidney disease, diabetes, aortic stenosis, and calciphylaxis [48].

Vascular calcification, particularly in the coronary arteries, significantly influences the trajectory and outcome of myocardial infarction (MI) [170]. Coronary artery calcification (CAC) is a key marker of plaque burden, as elevated CAC, often identified by computed tomography, substantially increases the risk of future MI or coronary heart disease mortality. Although statins effectively attenuate overall plaque growth and subsequent events, they paradoxically accelerate CAC progression, raising difficulties in treatment monitoring [171]. This unique response of statins contrasts with that of other cholesterol-lowering drugs, such as PCSK9 inhibitors, which show less impact on CAC progression, especially when used concomitantly with statins [172]. Furthermore, during coronary stenting for acute MI, severe and moderate calcification in culprit lesions significantly increases complications such as thrombosis or restenosis, highlighting the complexities imposed by calcification in clinical interventions for MI [173]. Therefore, it is imperative to understand the relationship between vascular calcification, CAC, and treatment responses to optimize risk assessment, guide therapeutic decisions, and improve outcomes after MI.

Vascular calcification, especially in the intracranial, coronary, and carotid arteries, significantly influences stroke risk, prognosis, and response to treatment [174]. Vascular calcification also affects cerebral hemodynamics and contributes to small vessel disease [175]. Recent studies exploring intracranial artery calcification (IAC) to predict stroke occurrence and post-stroke mortality revealed that patients with IAC face an increased risk of stroke and recurrence but not necessarily post-stroke mortality [176]. Despite the frequency and detectability of IAC on computed tomography angiography (CTA), its role as a prognostic tool for stroke risk or recurrence remains limited. Although it is recognized that atherosclerosis in intracranial arteries frequently contributes to stroke, the possible predictive value of IAC on stroke incidence, recurrence, and response to treatment of acute ischemic stroke is an area under exploration that requires further investigation.

Vascular calcification is highly prevalent among people with chronic kidney disease (CKD), increasing the risk of cardiovascular morbidity and mortality. Its development involves an interaction between altered mineral balance, chronic inflammation, and cellular responses of VSMCs, macrophages, and ECs [177]. CKD risk factors accelerate and initiate calcification earlier, often earlier than in the general population [178]. Alterations in mineral metabolism, especially calcium and phosphorus homeostasis, play a central role in the initiation and promotion of CV, as observed in clinical and experimental studies [179]. Low vitamin K levels lead to inactive forms of MGP, a calcification inhibitor, which is associated with CV acceleration.

Vascular calcification is related to diabetes mellitus (DM), in which several signaling pathways, such as TNF-α, and ILs, contribute to the development of vascular calcification [180]. Despite advances in antidiabetic drugs, their effects on the regression of vascular calcification remain unexplored. Similarly, in type 2 diabetes mellitus (T2DM), there is a strong association with vascular calcification driven by the OPG/RANKL/TRAIL system, which is normally involved in bone remodeling [181]. RANKL promotes vascular calcification, while OPG acts as a counterbalance, deviating from its functions in bone metabolism. Arterial calcification, prevalent in metabolic syndrome and diabetes, impairs vessel function and increases the risk of adverse outcomes [182]. Preclinical models point to BMP–Wnt signaling and endothelial–mesenchymal transition guiding arterial calcification in these conditions. Furthermore, elevated levels of dp-ucMGP, an inactive form of vitamin K-associated MGP, are associated with below-knee arterial calcification in patients with T2DM, warranting further exploration of its clinical significance and possible reversibility [183].

The echocardiographic examination is often used to establish the diagnosis of aortic stenosis (AS), which provides a wealth of information on the structure of the heart valve and blood flow characteristics [184]. Most patients are referred for echocardiography because of the appearance of symptoms such as dyspnea, angina pectoris, syncope, and dizziness, or because a systolic murmur is auscultated.

The occlusion of blood arteries in the dermis and subcutaneous fat causes calciphylaxis, also known as calcific uremic arteriolopathy, which is a cutaneous ischemic infarction that evolves into ulcerative lesions at risk of superinfection and sepsis [185]. Calciphylaxis is extremely debilitating due to severe pain and predisposition to infection; the annual mortality rate ranges from 40% to 80% [186]. Primarily, calciphylaxis is a condition of renal failure, and most patients are on or about to receive dialysis [187]. When a patient with end-stage renal failure presents with painful indurated plaques or ulcers on the belly and/or legs, the diagnosis can be determined solely from clinical considerations [188]. Physicians with expertise in nephrology, dermatology, plastic surgery, nutrition, and wound care are needed to treat calciphylaxis in a multidisciplinary fashion.

## 5. Translational Opportunities

### 5.1. Biomarkers of Vascular Calcification

The list of proposed biomarkers of vascular calcification is so extensive that it includes MGP, osteoprotegerin, bone morphogenetic proteins, fetuin-A, fibroblast growth factor 23, osteocalcin, osteopontin, osteonectin, sclerostin, pyrophosphate, Smads, fibrillin-1 and carbonic anhydrase II [189,190]. Therefore, we will review a few of them in the next section.

Fetuin-A is a circulating negatively charged protein, known as α2-Heremans–Schmid glycoprotein, that is produced by the liver and functions as an important local and systemic inhibitor of vascular calcification. It eliminates ectopic vascular calcification through several mechanisms, including the prevention of the growth of hydroxyapatite crystals outside cells and reducing OS and inflammation [191]. Fetuin-knockout mice showed severe and lethal extraosseous calcification in the heart, lungs, kidneys, and skin after treatment with a diet rich in vitamin D and phosphorus, confirming its crucial role as an inhibitor of calcification. It is intriguing to speculate that the measurement of serum fetuin-mineral complexes may function as a potential biomarker of ectopic calcification in addition to serum and hepatic fetuin-A levels [192]. Also, MGP is a vitamin K-dependent protein that is expressed locally by VSMCs and is considered inhibitory of vascular calcification. However, its corresponding inactive form, the uncarboxylated MGP (ucMGP), accumulates at sites of calcification [128,193]. Moreover, vitamin K deficiency, commonly found in CKD, is associated with higher levels of ucMGP. Therefore, the regulation of MGP is involved in the pathogenesis of vascular calcification and may be employed as a useful biomarker for risk assessment [127].

Smad proteins are vital for signal transduction from the receptor to the nucleus within the cell when the type I receptor is activated. The BMP-2/Smad signaling pathway is crucial for the osteoblastic differentiation of VSMCs, resulting in vascular calcification [164]. Osteoblast development is inhibited by a reduction in Smad11/5/8 expression when the BMP-2 signaling pathway is inhibited [194].

Fibrillin-1 is a 350 kDa glycoprotein rich in cysteines that produces elastic fibers and microfibrils in connective tissue. The flexibility of arterial walls is facilitated by elastic fibers, and vessel diseases are a consequence of the rupture of these fibers [195]. The control of elastic fiber homeostasis and cellular repair, two processes involved in matrix remodeling, depend on FBN-1 [196]. It is likely that FBN-1 is involved in VC through these pathways and is possibly a therapeutic target.

Two phosphate ions combine to generate PPi, which are tiny molecules that attach to hydroxyapatite and prevent further crystallization. They are believed to be potent inhibitors of medial vascular calcification. VSMCs secrete PPi. Three factors regulate local PPi concentrations: ectonucleotide pyrophosphatase phosphodiesterase (ENPP1), the multi-pass transmembrane protein encoded by the progressive ankylosis locus (ANK), and nonspecific tissue alkaline phosphatase (TNAP) [197]. The limiting enzyme ENPP1 controls the intracellular production of PPi, whereas ANK regulates the proper transport of PPi out of the cell. TNAP breaks down excess extracellular PPi into phosphate ions, which aids local defense against vascular calcification [198]. In humans, severe calcification, heart failure, and early mortality in the arteries of neonates and infants are the hallmarks of a debilitating disease known as generalized arterial calcification of infancy [199]. Mutations in ENPP1 induce the disease and cause its dysfunction.

FGF-23 is a protein that has 251 amino acids, a molecular weight of 32 kDa, and two distinct regions. Bone osteocytes release FGF-23 in response to an increase in dietary phosphate load, resulting in phosphaturia and a decrease in calcitriol levels [200]. For FGF-23 to be activated, it must bind its receptor and the co-receptor Klotho, which facilitates the binding of FGF-23 to FGFR [201]. Both FGF-23 and Klotho are important players in the pathophysiology of vascular calcification complications in CKD and can be used as early biomarkers as well as potential targets for vascular calcification and CKD therapy.

Carbonic anhydrase II (CA II) is a class of zinc-containing proteins that catalyze the reversible conversion of carbon dioxide to bicarbonate. It plays a role in gluconeogenesis, lipogenesis, osteoclast differentiation, acid–base balance, and volume contraction in humans. Proton generation in osteoclasts is facilitated by CA II, which in turn causes the acidification of resorption lacunae and ultimately dissolves bone. Macrophages also show significant levels of CA II expression [202]. In a genome-wide microarray analysis investigating the differential transcriptional pro-life for vascular calcification, CA II overexpression was found in human atheroma plaques compared with normal arterial tissue from the same individual [203]. Compared with normal tissue, atheroma plaque was found to overexpress CA II by a factor greater than 1.7 [68]. In conclusion, given that vascular calcification is linked to CA II production, this enzyme can be employed both as a potential therapeutic target and as a biomarker of calcification.

In the case of patients with CKD, serum calciprotein particles and serum calcification propensity are hallmarks of vascular calcification [204]. Serum calciprotein particles (CPPs) are colloidal nanoparticles composed of a combination of proteins, including fetuin-A, albumin, and Gla-rich protein (GRP), and calcium phosphates. We can distinguish two differentiated states regarding their level of maturation and aggregation: the primary CPPs (CPP I), which are small and spherical, and the secondary CPPs (CPP II), which are larger and have a damaging needle-shaped structure [205,206]. The serum calcification propensity is the intrinsic ability of serum to facilitate or inhibit the precipitation of calcium phosphate complexes. It is quantified by a recently developed in vitro assay termed T_50_ based on the half-transformation time from CPP I to CPP II. T50 is currently employed in research projects only. However, T_50_ is associated with cardiovascular events, mortality, and kidney disease progression, underscoring its importance as a prognostic marker, as a potentially therapeutic target, and as a management parameter of vascular calcification in patients with CKD, including those undergoing hemodialysis [207]. The presence of vascular calcification contributes significantly to increased mortality rates in patients with CKD due to cardiovascular complications, making these biomarkers of high clinical importance.

Lastly, PET-MDCT is a practical and repeatable technique that combines the anatomical images of MDCT with the molecular images of PET, which is used to identify biomarkers of aortic valve biology and flow patterns [208,209]. PET-MDCT measurements of valvular ^18^F-sodium fluoride (^18^F-NaF) uptake serve as a marker of the active mineralization process taking place within the valve.

### 5.2. Therapeutic Approaches

Modulation of the main regulators of vascular calcification is the basis of vascular calcification treatment. However, effective drugs or non-drug therapies against vascular calcification have not yet been found due to the complex underlying molecular regulation. The options better studied try to reduce the amount of calcium consumed (Table 1). Most of these therapeutic alternatives are being studied in clinical settings.

Elevated phosphate values are indicative of vascular calcification. Phosphate binders have been shown to reduce serum phosphorus levels by decreasing FGF23, which accelerates phosphorus excretion and prevents vascular calcification. Compared to calcium phosphate binders, a recent randomized study showed that the phosphate binder sevelamer reduced mortality more in elderly hemodialysis patients [210,211].

Ppi analogs and bisphosphonates have been used to treat osteoporosis by interfering with hydroxyapatite nucleation and development. In more recent research, when pamidronate and etidronate were administrated at the same dose, uremic mice also demonstrated the prevention of vascular calcification independent of bone resorption [212,213]. Exactly how bisphosphonates work is currently unknown; these drugs have different effects in different people, and there is a lack of information on patient safety with long-term use.

Vitamin K supplementation is necessary to maintain balance in calcium formation and blood clotting. Menaquinones (vitamin K2) and phylloquinone (vitamin K1) are the two naturally occurring forms of vitamin K. According to previously published research, the very active process of vascular calcification is partially controlled and prevented by MGP, which in turn is triggered by the carboxylation of glutamic acid residues in hemodialysis patients who are dependent of vitamin K [214,215]. Oral vitamin K2 administration to hemodialysis patients reduced serum uc-MGP levels but did not influence the progression of aortic calcification.

Sodium thiosulphate (NaTS) functions as an antioxidant and chelating agent, it is used as a treatment for cyanide poisoning and as a preventive measure against cisplatin toxicity. Compared to calcium oxalate or calcium phosphate, NaTS can chelate calcium to create very soluble complexes in the human body. It has been possible to treat individuals with nephrolithiasis, vascular calcification, and skin necrosis with NaTS [216,217]. Although clinical data are scarce, it is hypothesized that the antioxidant activity may function as an adjuvant in repairing damaged endothelial cells.

To evaluate the effects of drugs that allosterically activate calcium-sensing receptors to mimic the impact of calcium on cells, clinical studies on calcimimetics are ongoing. Regarding vitamin D and calcimimetic-guided therapy for the treatment of mineral bone disease and life-limiting cardiovascular disease in the CKD population, a few sophisticated and well-planned controlled studies are available. It has been documented that calcimimetics such as R-568 and AMG-641 can inhibit vascular calcification [218]. This action is also related to the increased sensitivity of calcium-sensing receptors to extracellular calcium [219]. However, due to the evidence, their ability to prevent calcification remains in question.

The natural substance myo-inositol hexaphosphate (IP6), which is an endogenous intracellular phosphate storage molecule in plants and mammals, prevents the production and development of hydroxyapatite microcrystals without directly affecting blood levels of calcium and phosphate [220,221]. Due to its short plasma half-life (minutes) and poor oral absorption, its infusion is administered intravenously.

The human monoclonal antibody denosumab can bind to and inhibit human RANKL like the inherent bone-protective properties of OPG. Clinical studies with patients with breast cancer or bone metastases from multiple myeloma demonstrated that an injection of denosumab suppressed bone turnover markers rapidly and persistently. Based on calcium measurements, research in human RANKL knock-in (huRANKL-KI) mice revealed that huRANKL-KI mice treated with prednisolone had a 50% decrease in aortic calcium deposits when treated with denosumab [222,223].

**Table 1:** Main therapeutic opportunities against different targets of vascular calcification. Ppi: inorganic pyrophosphate, MGP: matrix Gla protein, NaTS: sodium thiosulphate, IP6: myo-inositol hexaphosphate, RANKL: receptor activator of nuclear factor κB ligand.

Compound	Target	Mechanism of Action	References
Sevelamer	Phosphate	Phosphate binder	[210,211]
Ppi analogs	Hydroxyapatite	Disruption of hydroxyapatite nucleation	[212,213]
Vitamin K2	MGP	Activation of MGP	[214]
NaTS	Calcium	Chelation of calcium	[216]
Calcimimetics	Calcium-sensing receptors	Increase the sensitivity of calcium-sensing receptors	[219]
IP6	Hydroxyapatite	Disruption of hydroxyapatite nucleation	[220]
Denosumab	RANKL	Inhibition of RANKL signaling	[223]

## 6. Conclusions

Cellular interactions, signaling pathways, and molecular mediators are involved in vascular calcification. Cells such as VSMCs, ECs, and macrophages play critical roles in vascular calcification through phenotypic changes and the expression of mineralization-related proteins. Calcium, Wnt, BMP, and Notch signaling pathways are involved in promoting or inhibiting calcification in a complex interplay, suggesting possible therapeutic targets to mitigate vascular calcification. Some studies, such as those investigating vitamin K2 in hemodialysis patients or the interaction between intimal and medial calcifications, provide clinical information. However, gaps remain, such as the limited effect of certain interventions on disease progression. Despite advances, the mechanisms driving vascular calcification remain incompletely understood. Future research should deepen into molecular networking and cellular interactions to identify specific therapeutic targets and interventions.

## References

[1] Proudfoot D. (2019). Calcium Signaling and Tissue Calcification. Cold Spring Harb. Perspect. Biol..

[2] De Leon-Oliva D., Barrena-Blázquez S., Jiménez-Álvarez L., Fraile-Martinez O., García-Montero C., López-González L., Torres-carranza D., García-Puente L.M., Carranza S.T., Álvarez-Mon M.Á. (2023). The RANK–RANKL–OPG System: A Multifaceted Regulator of Homeostasis, Immunity, and Cancer. Medicina.

[3] Murshed M. (2018). Mechanism of Bone Mineralization. Cold Spring Harb. Perspect. Med..

[4] De Leon-Oliva D., Boaru D.L., Perez-Exposito R.E., Fraile-Martinez O., García-Montero C., Díaz R., García-Honduvilla N., Lopez-Gonzalez L., Alvarez-Mon M., Saz J.V. (2023). Advanced Hydrogel-Based Strategies for Enhanced Bone and Cartilage Regeneration: A Comprehensive Review. Gels.

[5] Fraile-Martínez O., García-Montero C., Coca A., Álvarez-Mon M.A., Monserrat J., Gómez-lahoz A.M., Coca S., Álvarez-mon M., Acero J., Bujan J. (2021). Applications of Polymeric Composites in Bone Tissue Engineering and Jawbone Regeneration. Polymers.

[6] Sutton N.R., Malhotra R., Hilaire C.S., Aikawa E., Blumenthal R.S., Gackenbach G., Goyal P., Johnson A., Nigwekar S.U., Shanahan C.M. (2023). Molecular Mechanisms of Vascular Health: Insights From Vascular Aging and Calcification. Arterioscler. Thromb. Vasc. Biol..

[7] Vidavsky N., Kunitake J.A.M.R., Estroff L.A. (2021). Multiple Pathways for Pathological Calcification in the Human Body. Adv. Healthc. Mater..

[8] Demer L.L., Tintut Y. (2014). Inflammatory, Metabolic, and Genetic Mechanisms of Vascular Calcification. Arterioscler. Thromb. Vasc. Biol..

[9] Ortolani F., Rigonat L., Bonetti A., Contin M., Tubaro F., Rattazzi M., Marchini M. (2010). Pro-Calcific Responses by Aortic Valve Interstitial Cells in a Novel in Vitro Model Simulating Dystrophic Calcification. Ital. J. Anat. Embryol..

[10] Senba M., Kawai K., Mori N. (2012). Pathogenesis of Metastatic Calcification and Acute Pancreatitis in Adult T-Cell Leukemia under Hypercalcemic State. Leuk. Res. Treat..

[11] Kim H.Y., Park J.H., Lee J.B., Kim S.J. (2017). A Case of Dystrophic Calcification in the Masseter Muscle. Maxillofac. Plast. Reconstr. Surg..

[12] Bonetti A., Della Mora A., Contin M., Tubaro F., Marchini M., Ortolani F. (2012). Ultrastructural and Spectrophotometric Study on the Effects of Putative Triggers on Aortic Valve Interstitial Cells in In Vitro Models Simulating Metastatic Calcification. Anat. Rec..

[13] Valenzuela A., Chung L. (2015). Calcinosis: Pathophysiology and Management. Curr. Opin. Rheumatol..

[14] Sawke G.K., Rai T., Sawke N. (2016). Iatrogenic Calcinosis Cutis: A Rare Cytological Diagnosis. J. Cytol..

[15] Ibragimova A.G., Stanishevskiy Y.M., Plakkhin A.M., Zubko A.V., Darvish N.A., Koassary A.K., Shindyapina A.V. (2023). Comparative Analysis of Calcified Soft Tissues Revealed Shared Deregulated Pathways. Front. Aging Neurosci..

[16] Ortega M.A., Saez M.Á., Asúnsolo Á., Romero B., Bravo C., Coca S., Sainz F., Álvarez-Mon M., Buján J., García-Honduvilla N. (2019). Upregulation of VEGF and PEDF in Placentas of Women with Lower Extremity Venous Insufficiency during Pregnancy and Its Implication in Villous Calcification. BioMed Res. Int..

[17] Kim S., Tran T.X.M., Song H., Park B. (2022). Microcalcifications, Mammographic Breast Density, and Risk of Breast Cancer: A Cohort Study. Breast Cancer Res..

[18] Monfrini E., Arienti F., Rinchetti P., Lotti F., Riboldi G.M. (2023). Brain Calcifications: Genetic, Molecular, and Clinical Aspects. Int. J. Mol. Sci..

[19] Wyatt C.M., Drueke T.B. (2017). Vascular Calcification in Chronic Kidney Disease: Here to Stay?. Kidney Int..

[20] Kosuru V., Mohammed A., Kapoor R., Jhaveri K., Medepalli V., Mulloy L., Padala S.A. (2020). Metastatic Calcinosis of Gastric Mucosa. J. Investig. Med. High Impact Case Rep..

[21] Kim M.-S., Kim I.-W., Lee S., Shin S.-J. (2020). Diagnosis and Treatment of Calcific Tendinitis of the Shoulder. Clin. Shoulder Elb..

[22] Jarjou’i A., Bogot N., Kalak G., Chen-Shuali C., Rokach A., Izbicki G., Arish N. (2021). Diffuse Pulmonary Calcifications: A Case Series and Review of Literature. Respirol. Case Rep..

[23] Poloni L.N., Ward M.D. (2014). The Materials Science of Pathological Crystals. Chem. Mater..

[24] Bazin D., Daudon M., Combes C., Rey C. (2012). Characterization and Some Physicochemical Aspects of Pathological Microcalcifications. Chem. Rev..

[25] Bonetti A., Contin M., Marchini M., Ortolani F. (2023). Ultrastructural and Immunohistochemical Detection of Hydroxyapatite Nucleating Role by RRNA and Nuclear Chromatin Derivatives in Aortic Valve Calcification: In Vitro and In Vivo Pro-Calcific Animal Models and Actual Calcific Disease in Humans. Int. J. Mol. Sci..

[26] Demer L.L., Tintut Y. (2008). Vascular Calcification: Pathobiology of a Multifaceted Disease. Circulation.

[27] Bonetti A., Marchini M., Ortolani F. (2019). Ectopic Mineralization in Heart Valves: New Insights from in Vivo and in Vitro Procalcific Models and Promising Perspectives on Noncalcifiable Bioengineered Valves. J. Thorac. Dis..

[28] Wu M., Rementer C., Giachelli C.M. (2013). Vascular Calcification: An Update on Mechanisms and Challenges in Treatment. Calcif. Tissue Int..

[29] Kuczumow A., Gorzelak M., Kosiński J., Lasota A., Blicharski T., Gągała J., Nowak J., Jarzębski M., Jabłoński M. (2022). Hierarchy of Bioapatites. Int. J. Mol. Sci..

[30] Chen Y., Zhao X., Wu H. (2020). Arterial Stiffness: A Focus on Vascular Calcification and Its Link to Bone Mineralization. Arterioscler. Thromb. Vasc. Biol..

[31] You A.Y.F., Bergholt M.S., St-Pierre J.P., Kit-Anan W., Pence I.J., Chester A.H., Yacoub M.H., Bertazzo S., Stevens M.M. (2017). Raman Spectroscopy Imaging Reveals Interplay between Atherosclerosis and Medial Calcification in the Human Aorta. Sci. Adv..

[32] Lanzer P., Hannan F.M., Lanzer J.D., Janzen J., Raggi P., Furniss D., Schuchardt M., Thakker R., Fok P.W., Saez-Rodriguez J. (2021). Medial Arterial Calcification: JACC State-of-the-Art Review. J. Am. Coll. Cardiol..

[33] Kim T.I., Guzman R.J. (2023). Medial Artery Calcification in Peripheral Artery Disease. Front. Cardiovasc. Med..

[34] Zhu D., Mackenzie N.C.W., Farquharson C., MacRae V.E. (2012). Mechanisms and Clinical Consequences of Vascular Calcification. Front. Endocrinol..

[35] Van den Bergh G., Opdebeeck B., D’Haese P.C., Verhulst A. (2019). The Vicious Cycle of Arterial Stiffness and Arterial Media Calcification. Trends Mol. Med..

[36] Lerman D.A., Prasad S., Alotti N. (2015). Calcific Aortic Valve Disease: Molecular Mechanisms and Therapeutic Approaches. Eur. Cardiol..

[37] Moncla L.H.M., Briend M., Bossé Y., Mathieu P. (2023). Calcific Aortic Valve Disease: Mechanisms, Prevention and Treatment. Nat. Rev. Cardiol..

[38] Kraler S., Blaser M.C., Aikawa E., Camici G.G., Lüscher T.F. (2022). Calcific Aortic Valve Disease: From Molecular and Cellularmechanisms to Medical Therapy. Eur. Heart J..

[39] Pawade T., Sheth T., Guzzetti E., Dweck M.R., Clavel M.A. (2019). Why and How to Measure Aortic Valve Calcification in Patients With Aortic Stenosis. JACC Cardiovasc. Imaging.

[40] Chen H.Y., Engert J.C., Thanassoulis G. (2019). Risk Factors for Valvular Calcification. Curr. Opin. Endocrinol. Diabetes Obes..

[41] Ahn B.Y., Jeong Y., Kim S., Zhang Y., Kim S.W., Leem Y.E., Kang J.S. (2023). Cdon Suppresses Vascular Smooth Muscle Calcification via Repression of the Wnt/Runx2 Axis. Exp. Mol. Med..

[42] Yang P., Troncone L., Augur Z.M., Kim S.S.J., McNeil M.E., Yu P.B. (2020). The Role of Bone Morphogenetic Protein Signaling in Vascular Calcification. Bone.

[43] Semenova D., Kostina A., Irtyuga O., Moiseeva O., Malashicheva A. (2019). The Mechanisms of Notch-Dependent Aortic Valve Calcification. Struct. Heart.

[44] Pustlauk W., Westhoff T.H., Claeys L., Roch T., Geißler S., Babel N. (2020). Induced Osteogenic Differentiation of Human Smooth Muscle Cells as a Model of Vascular Calcification. Sci. Rep..

[45] Cui L., Houston D.A., Farquharson C., MacRae V.E. (2016). Characterisation of Matrix Vesicles in Skeletal and Soft Tissue Mineralisation. Bone.

[46] Jiang W., Zhang Z., Li Y., Chen C., Yang H., Lin Q., Hu M., Qin X. (2021). The Cell Origin and Role of Osteoclastogenesis and Osteoblastogenesis in Vascular Calcification. Front. Cardiovasc. Med..

[47] Yap C., Mieremet A., De Vries C.J.M., Micha D., De Waard V. (2021). Six Shades of Vascular Smooth Muscle Cells Illuminated by KLF4 (Krüppel-Like Factor 4). Arterioscler. Thromb. Vasc. Biol..

[48] Tintut Y., Honda H.M., Demer L.L. (2021). Biomolecules Orchestrating Cardiovascular Calcification. Biomolecules.

[49] Jiang H., Li L., Zhang L., Zang G., Sun Z., Wang Z. (2022). Role of Endothelial Cells in Vascular Calcification. Front. Cardiovasc. Med..

[50] Wylie-Sears J., Aikawa E., Levine R.A., Yang J.H., Bischoff J. (2011). Mitral Valve Endothelial Cells with Osteogenic Differentiation Potential. Arterioscler. Thromb. Vasc. Biol..

[51] Wen Y.D., Wang H., Zhu Y.Z. (2018). The Drug Developments of Hydrogen Sulfide on Cardiovascular Disease. Oxid. Med. Cell. Longev..

[52] Kim S.H.L., Lee S.S., Kim I., Kwon J., Kwon S., Bae T., Hur J., Lee H., Hwang N.S. (2020). Ectopic Transient Overexpression of OCT-4 Facilitates BMP4-Induced Osteogenic Transdifferentiation of Human Umbilical Vein Endothelial Cells. J. Tissue Eng..

[53] Durham A.L., Speer M.Y., Scatena M., Giachelli C.M., Shanahan C.M. (2018). Role of Smooth Muscle Cells in Vascular Calcification: Implications in Atherosclerosis and Arterial Stiffness. Cardiovasc. Res..

[54] Voelkl J., Lang F., Eckardt K.U., Amann K., Kuro-o M., Pasch A., Pieske B., Alesutan I. (2019). Signaling Pathways Involved in Vascular Smooth Muscle Cell Calcification during Hyperphosphatemia. Cell. Mol. Life Sci..

[55] Rong J.X., Shapiro M., Trogan E., Fisher E.A. (2003). Transdifferentiation of Mouse Aortic Smooth Muscle Cells to a Macrophage-like State after Cholesterol Loading. Proc. Natl. Acad. Sci. USA.

[56] Lin X., Shan S.K., Xu F., Zhong J.Y., Wu F., Duan J.Y., Guo B., Li F.X.Z., Wang Y., Zheng M.H. (2022). The Crosstalk between Endothelial Cells and Vascular Smooth Muscle Cells Aggravates High Phosphorus-Induced Arterial Calcification. Cell Death Dis..

[57] Ye G.J.C., Nesmith A.P., Parker K.K. (2014). The Role of Mechanotransduction on Vascular Smooth Muscle Myocytes Cytoskeleton and Contractile Function. Anat. Rec..

[58] Gomel M.A., Lee R., Grande-Allen K.J. (2019). Comparing the Role of Mechanical Forces in Vascular and Valvular Calcification Progression. Front. Cardiovasc. Med..

[59] Balogh E., Tóth A., Méhes G., Trencsényi G., Paragh G., Jeney V. (2019). Hypoxia Triggers Osteochondrogenic Differentiation of Vascular Smooth Muscle Cells in an HIF-1 (Hypoxia-Inducible Factor 1)-Dependent and Reactive Oxygen Species-Dependent Manner. Arterioscler. Thromb. Vasc. Biol..

[60] Csiki D.M., Ababneh H., Tóth A., Lente G., Szöőr Á., Tóth A., Fillér C., Juhász T., Nagy B., Balogh E. (2023). Hypoxia-Inducible Factor Activation Promotes Osteogenic Transition of Valve Interstitial Cells and Accelerates Aortic Valve Calcification in a Mice Model of Chronic Kidney Disease. Front. Cardiovasc. Med..

[61] Huang X., Akgün E.E., Mehmood K., Zhang H., Tang Z., Li Y. (2022). Mechanism of Hypoxia-Mediated Smooth Muscle Cell Proliferation Leading to Vascular Remodeling. BioMed Res. Int..

[62] Rangrez A.Y., M’Baya-Moutoula E., Metzinger-Le Meuth V., Hénaut L., Djelouat M.S.E.I., Benchitrit J., Massy Z.A., Metzinger L. (2012). Inorganic Phosphate Accelerates the Migration of Vascular Smooth Muscle Cells: Evidence for the Involvement of MiR-223. PLoS ONE.

[63] Shimokado A., Sun Y., Nakanishi M., Sato F., Oikawa K., Akasaka T., Muragaki Y. (2014). Smad3 Plays an Inhibitory Role in Phosphate-Induced Vascular Smooth Muscle Cell Calcification. Exp. Mol. Pathol..

[64] Metzinger-Le Meuth V., Metzinger L. (2019). MiR-223 and Other MiRNA’s Evaluation in Chronic Kidney Disease: Innovative Biomarkers and Therapeutic Tools. Non-Coding RNA Res..

[65] Aghagolzadeh P., Bachtler M., Bijarnia R., Jackson C., Smith E.R., Odermatt A., Radpour R., Pasch A. (2016). Calcification of Vascular Smooth Muscle Cells Is Induced by Secondary Calciprotein Particles and Enhanced by Tumor Necrosis Factor-α. Atherosclerosis.

[66] Luong T.T.D., Estepa M., Boehme B., Pieske B., Lang F., Eckardt K.U., Voelkl J., Alesutan I. (2019). Inhibition of Vascular Smooth Muscle Cell Calcification by Vasorin through Interference with TGFβ1 Signaling. Cell. Signal..

[67] Cao J., Chen L., Zhong X., Shen Y., Gao Y., Chen Q., Zu X., Liu J. (2020). MiR32-5p Promoted Vascular Smooth Muscle Cell Calcification by Upregulating TNFα in the Microenvironment. BMC Immunol..

[68] Song X., Song Y., Ma Q., Fang K., Chang X. (2023). M1-Type Macrophages Secrete TNF-α to Stimulate Vascular Calcification by Upregulating CA1 and CA2 Expression in VSMCs. J. Inflamm. Res..

[69] Li Y., Sun Z., Zhang L., Yan J., Shao C., Jing L., Li L., Wang Z. (2020). Role of Macrophages in the Progression and Regression of Vascular Calcification. Front. Pharmacol..

[70] Waring O.J., Skenteris N.T., Biessen E.A.L., Donners M.M.P.C. (2022). Two-Faced Janus: The Dual Role of Macrophages in Atherosclerotic Calcification. Cardiovasc. Res..

[71] Chinetti-Gbaguidi G., Colin S., Staels B. (2015). Macrophage Subsets in Atherosclerosis. Nat. Rev. Cardiol..

[72] Li M., Wang Z.W., Fang L.J., Cheng S.Q., Wang X., Liu N.F. (2022). Programmed Cell Death in Atherosclerosis and Vascular Calcification. Cell Death Dis..

[73] Hénaut L., Candellier A., Boudot C., Grissi M., Mentaverri R., Choukroun G., Brazier M., Kamel S., Massy Z.A. (2019). New Insights into the Roles of Monocytes/Macrophages in Cardiovascular Calcification Associated with Chronic Kidney Disease. Toxins.

[74] Proudfoot D., Skepper J.N., Hegyi L., Bennett M.R., Shanahan C.M., Weissberg P.L. (2000). Apoptosis Regulates Human Vascular Calcification in Vitro: Evidence for Initiation of Vascular Calcification by Apoptotic Bodies. Circ. Res..

[75] Proudfoot D., Skepper J.N., Hegyi L., Farzaneh-Far A., Shanahan C.M., Weissberg P.L. (2001). The Role of Apoptosis in the Initiation of Vascular Calcification. Z. Kardiol..

[76] Doherty M.J., Ashton B.A., Walsh S., Beresford J.N., Grant M.E., Canfield A.E. (1998). Vascular Pericytes Express Osteogenic Potential in Vitro and in Vivo. J. Bone Miner. Res..

[77] Canfield A.E., Doherty M.J., Wood A.C., Farrington C., Ashton B., Begum N., Harvey B., Poole A., Grant M.E., Boot-Handford R.P. (2000). Role of Pericytes in Vascular Calcification: A Review. Z. Kardiol..

[78] Davaine J.M., Quillard T., Brion R., Lapérine O., Guyomarch B., Merlini T., Chatelais M., Guilbaud F., Brennan M.Á., Charrier C. (2014). Osteoprotegerin, Pericytes and Bone-like Vascular Calcification Are Associated with Carotid Plaque Stability. PLoS ONE.

[79] Hortells L., Sur S., Hilaire C.S. (2018). Cell Phenotype Transitions in Cardiovascular Calcification. Front. Cardiovasc. Med..

[80] Simionescu A., Simionescu D.T., Vyavahare N.R. (2007). Osteogenic Responses in Fibroblasts Activated by Elastin Degradation Products and Transforming Growth Factor-Β1: Role of Myofibroblasts in Vascular Calcification. Am. J. Pathol..

[81] Li W., Su S., Chen J., Ma H., Xiang M. (2021). Emerging Roles of Fibroblasts in Cardiovascular Calcification. J. Cell. Mol. Med..

[82] Bundy K., Boone J., Simpson C.L.S. (2021). Wnt Signaling in Vascular Calcification. Front. Cardiovasc. Med..

[83] Shimizu T., Tanaka T., Iso T., Doi H., Sato H., Kawai-Kowase K., Arai M., Kurabayashi M. (2009). Notch Signaling Induces Osteogenic Differentiation and Mineralization of Vascular Smooth Muscle Cells: Role of Msx2 Gene Induction via Notch-RBP-Jk Signaling. Arterioscler. Thromb. Vasc. Biol..

[84] Zhao X.-K., Zhu M.-M., Wang S.-N., Zhang T.-T., Wei X.-N., Wang C.-Y., Zheng J., Zhu W.-Y., Jiang M.-X., Xu S.-W. (2023). Transcription Factor 21 Accelerates Vascular Calcification in Mice by Activating the IL-6/STAT3 Signaling Pathway and the Interplay between VSMCs and ECs. Acta Pharmacol. Sin..

[85] Donate-Correa J., Martín-Núñez E., Hernández-Carballo C., Ferri C., Tagua V.G., Delgado-Molinos A., López-Castillo Á., Rodríguez-Ramos S., Cerro-López P., López-Tarruella V.C. (2019). Fibroblast Growth Factor 23 Expression in Human Calcified Vascular Tissues. Aging.

[86] Chen N.X., O’Neill K.D., Moe S.M. (2018). Matrix Vesicles Induce Calcification of Recipient Vascular Smooth Muscle Cells through Multiple Signaling Pathways. Kidney Int..

[87] Clapham D.E. (2007). Calcium Signaling. Cell.

[88] Bootman M.D., Bultynck G. (2020). Fundamentals of Cellular Calcium Signaling: A Primer. Cold Spring Harb. Perspect. Biol..

[89] Issa H., Hénaut L., Abdallah J.B., Boudot C., Lenglet G., Avondo C., Ibrik A., Caus T., Brazier M., Mentaverri R. (2019). Activation of the Calcium-Sensing Receptor in Human Valvular Interstitial Cells Promotes Calcification. J. Mol. Cell. Cardiol..

[90] Molostvov G., Hiemstra T.F., Fletcher S., Bland R., Zehnder D. (2015). Arterial Expression of the Calcium-Sensing Receptor Is Maintained by Physiological Pulsation and Protects against Calcification. PLoS ONE.

[91] Ma C., Gu R., Wang X., He S., Bai J., Zhang L., Zhang J., Li Q., Qu L., Xin W. (2020). CircRNA CDR1as Promotes Pulmonary Artery Smooth Muscle Cell Calcification by Upregulating CAMK2D and CNN3 via Sponging MiR-7-5p. Mol. Ther. Nucleic Acids.

[92] Li S.J., Kao Y.H., Chung C.C., Cheng W.L., Lin Y.K., Chen Y.J. (2021). Vascular Endothelial Growth Factor on Runt-Related Transcript Factor-2 in Aortic Valve Cells. Eur. J. Clin. Investig..

[93] Bonetti A., Allegri L., Baldan F., Contin M., Battistella C., Damante G., Marchini M., Ortolani F. (2020). Critical Involvement of Calcium-Dependent Cytosolic Phospholipase A2α in Aortic Valve Interstitial Cell Calcification. Int. J. Mol. Sci..

[94] Liu J., Xiao Q., Xiao J., Niu C., Li Y., Zhang X., Zhou Z., Shu G., Yin G. (2022). Wnt/β-Catenin Signalling: Function, Biological Mechanisms, and Therapeutic Opportunities. Signal Transduct. Target. Ther..

[95] Kang J.H., Kawano T., Murata M., Toita R. (2024). Vascular Calcification and Cellular Signaling Pathways as Potential Therapeutic Targets. Life Sci..

[96] Albanese I., Khan K., Barratt B., Al-Kindi H., Schwertani A. (2018). Atherosclerotic Calcification: Wnt Is the Hint. J. Am. Heart Assoc..

[97] Komiya Y., Habas R. (2008). Wnt Signal Transduction Pathways. Organogenesis.

[98] Albanese I., Yu B., Al-Kindi H., Barratt B., Ott L., Al-Refai M., De Varennes B., Shum-Tim D., Cerruti M., Gourgas O. (2017). Role of Noncanonical Wnt Signaling Pathway in Human Aortic Valve Calcification. Arterioscler. Thromb. Vasc. Biol..

[99] Sanchez-Duffhues G., Williams E., Goumans M.J., Heldin C.H., ten Dijke P. (2020). Bone Morphogenetic Protein Receptors: Structure, Function and Targeting by Selective Small Molecule Kinase Inhibitors. Bone.

[100] Niu Z., Su G., Li T., Yu H., Shen Y., Zhang D., Liu X. (2022). Vascular Calcification: New Insights Into BMP Type I Receptor, A. Front. Pharmacol..

[101] Morrell N.W., Bloch D.B., Ten Dijke P., Goumans M.J.T.H., Hata A., Smith J., Yu P.B., Bloch K.D. (2016). Targeting BMP Signalling in Cardiovascular Disease and Anaemia. Nat. Rev. Cardiol..

[102] Cai J., Pardali E., Sánchez-Duffhues G., Ten Dijke P. (2012). BMP Signaling in Vascular Diseases. FEBS Lett..

[103] Ortega M.A., Asúnsolo Á., Pekarek L., Alvarez-Mon M.A., Delforge A., Sáez M.A., Coca S., Sainz F., Álvarez-Mon M., Buján J. (2021). Histopathological Study of JNK in Venous Wall of Patients with Chronic Venous Insufficiency Related to Osteogenesis Process. Int. J. Med. Sci..

[104] Ye D., Liu Y., Pan H., Feng Y., Lu X., Gan L., Wan J., Ye J. (2023). Insights into Bone Morphogenetic Proteins in Cardiovascular Diseases. Front. Pharmacol..

[105] Zhang M., Sara J.D., Wang F.L., Liu L.P., Su L.X., Zhe J., Wu X., Liu J.H. (2015). Increased Plasma BMP-2 Levels Are Associated with Atherosclerosis Burden and Coronary Calcification in Type 2 Diabetic Patients. Cardiovasc. Diabetol..

[106] Panizo S., Cardus A., Encinas M., Parisi E., Valcheva P., López-Ongil S., Coll B., Fernandez E., Valdivielso J.M. (2009). RANKL Increases Vascular Smooth Muscle Cell Calcification through a Rank-Bmp4-Dependent Pathway. Circ. Res..

[107] Huang J., Pu Y., Zhang H., Xie L., He L., Zhang C.L., Cheng C.K., Huo Y., Wan S., Chen S. (2021). KLF2 Mediates the Suppressive Effect of Laminar Flow on Vascular Calcification by Inhibiting Endothelial BMP/SMAD1/5 Signaling. Circ. Res..

[108] Yung L.M., Sánchez-Duffhues G., Ten Dijke P., Yu P.B. (2015). Bone Morphogenetic Protein 6 and Oxidized Low-Density Lipoprotein Synergistically Recruit Osteogenic Differentiation in Endothelial Cells. Cardiovasc. Res..

[109] Davies M.R., Lund R.J., Hruska K.A. (2003). BMP-7 Is an Efficacious Treatment of Vascular Calcification in a Murine Model of Atherosclerosis and Chronic Renal Failure. J. Am. Soc. Nephrol..

[110] Lee C.-T., Kuo W.H., Tain Y.L., Wang Y., Lee W.C. (2022). Exogenous BMP7 Administration Attenuated Vascular Calcification and Improved Bone Disorders in Chronic Uremic Rats. Biochem. Biophys. Res. Commun..

[111] Rusanescu G., Weissleder R., Aikawa E. (2008). Notch Signaling in Cardiovascular Disease and Calcification. Curr. Cardiol. Rev..

[112] Wang Y., Fang Y., Lu P., Wu B., Zhou B. (2021). NOTCH Signaling in Aortic Valve Development and Calcific Aortic Valve Disease. Front. Cardiovasc. Med..

[113] Majumdar U., Manivannan S., Basu M., Ueyama Y., Blaser M.C., Cameron E., McDermott M.R., Lincoln J., Cole S.E., Wood S. (2021). Nitric Oxide Prevents Aortic Valve Calcification by S-Nitrosylation of USP9X to Activate NOTCH Signaling. Sci. Adv..

[114] Beazley K.E., Nurminsky D., Lima F., Gandhi C., Nurminskaya M.V. (2015). Wnt16 Attenuates TGFβ-Induced Chondrogenic Transformation in Vascular Smooth Muscle. Arterioscler. Thromb. Vasc. Biol..

[115] Lin X., Li S., Wang Y.J., Wang Y., Zhong J.Y., He J.Y., Cui X.J., Zhan J.K., Liu Y.S. (2019). Exosomal Notch3 from High Glucose-Stimulated Endothelial Cells Regulates Vascular Smooth Muscle Cells Calcification/Aging. Life Sci..

[116] White M.P., Theodoris C.V., Liu L., Collins W.J., Blue K.W., Lee J.H., Meng X., Robbins R.C., Ivey K.N., Srivastava D. (2015). NOTCH1 Regulates Matrix Gla Protein and Calcification Gene Networks in Human Valve Endothelium. J. Mol. Cell. Cardiol..

[117] Ouyang L., Zhang K., Chen J., Wang J., Huang H. (2018). Roles of Platelet-Derived Growth Factor in Vascular Calcification. J. Cell. Physiol..

[118] Shi Y., Wang S., Peng H., Lv Y., Li W., Cheng S., Liu J. (2019). Fibroblast Growth Factor 21 Attenuates Vascular Calcification by Alleviating Endoplasmic Reticulum Stress Mediated Apoptosis in Rats. Int. J. Biol. Sci..

[119] Olapoju S.O., Adejobi O.I., Le Thi X. (2020). Fibroblast Growth Factor 21; Review on Its Participation in Vascular Calcification Pathology. Vascul. Pharmacol..

[120] Sung D.C., Bowen C.J., Vaidya K.A., Zhou J., Chapurin N., Recknagel A., Zhou B., Chen J., Kotlikoff M., Butcher J.T. (2016). Cadherin-11 Overexpression Induces Extracellular Matrix Remodeling and Calcification in Mature Aortic Valves. Arterioscler. Thromb. Vasc. Biol..

[121] Vaidya K.A., Donnelly M.P., Mahmut A., Jang J.W., Gee T.W., Aibo M.A.I., Bossong R., Hall C., Samb S., Chen J. (2022). Rac1 Mediates Cadherin-11 Induced Cellular Pathogenic Processes in Aortic Valve Calcification. Cardiovasc. Pathol..

[122] Hutcheson J.D., Chen J., Sewell-Loftin M.K., Ryzhova L.M., Fisher C.I., Su Y.R., David Merryman W. (2013). Cadherin-11 Regulates Cell-Cell Tension Necessary for Calcific Nodule Formation by Valvular Myofibroblasts. Arterioscler. Thromb. Vasc. Biol..

[123] Bowler M.A., Bersi M.R., Ryzhova L.M., Jerrell R.J., Parekh A., Merryman W.D. (2018). Cadherin-11 as a Regulator of Valve Myofibroblast Mechanobiology. Am. J. Physiol. Heart Circ. Physiol..

[124] Radvar E., Griffanti G., Tsolaki E., Bertazzo S., Nazhat S.N., Addison O., Mata A., Shanahan C.M., Elsharkawy S. (2021). Engineered In Vitro Models for Pathological Calcification: Routes Toward Mechanistic Understanding. Adv. NanoBiomed Res..

[125] Bjørklund G., Svanberg E., Dadar M., Card D.J., Chirumbolo S., Harrington D.J., Aaseth J. (2020). The Role of Matrix Gla Protein (MGP) in Vascular Calcification. Curr. Med. Chem..

[126] Epstein M. (2016). Matrix Gla-Protein (MGP) Not Only Inhibits Calcification in Large Arteries But Also May Be Renoprotective: Connecting the Dots. EBioMedicine.

[127] Jaminon A.M.G., Dai L., Qureshi A.R., Evenepoel P., Ripsweden J., Söderberg M., Witasp A., Olauson H., Schurgers L.J., Stenvinkel P. (2020). Matrix Gla Protein Is an Independent Predictor of Both Intimal and Medial Vascular Calcification in Chronic Kidney Disease. Sci. Rep..

[128] Barrett H., O’Keeffe M., Kavanagh E., Walsh M., O’Connor E.M. (2018). Is Matrix Gla Protein Associated with Vascular Calcification? A Systematic Review. Nutrients.

[129] Ge Q., Ruan C.C., Ma Y., Tang X.F., Wu Q.H., Wang J.G., Zhu D.L., Gao P.J. (2017). Osteopontin Regulates Macrophage Activation and Osteoclast Formation in Hypertensive Patients with Vascular Calcification. Sci. Rep..

[130] Lok Z.S.Y., Lyle A.N. (2019). Osteopontin in Vascular Disease. Arterioscler. Thromb. Vasc. Biol..

[131] Van Campenhout A., Golledge J. (2009). Osteoprotegerin, Vascular Calcification and Atherosclerosis. Atherosclerosis.

[132] Zhou S., Fang X., Xin H., Li W., Qiu H., Guan S. (2013). Osteoprotegerin Inhibits Calcification of Vascular Smooth Muscle Cell via Down Regulation of the Notch1-RBP-Jκ/Msx2 Signaling Pathway. PLoS ONE.

[133] Dekker M., Waissi F., Silvis M.J.M., Bennekom J.V., Schoneveld A.H., de Winter R.J., Isgum I., Lessmann N., Velthuis B.K., Pasterkamp G. (2021). High Levels of Osteoprotegerin Are Associated with Coronary Artery Calcification in Patients Suspected of a Chronic Coronary Syndrome. Sci. Rep..

[134] Qin Z., Liao R., Xiong Y., Jiang L., Li J., Wang L., Han M., Sun S., Geng J., Yang Q. (2021). A Narrative Review of Exosomes in Vascular Calcification. Ann. Transl. Med..

[135] Zazzeroni L., Faggioli G., Pasquinelli G. (2018). Mechanisms of Arterial Calcification: The Role of Matrix Vesicles. Eur. J. Vasc. Endovasc. Surg..

[136] Li T., Yu H., Zhang D., Feng T., Miao M., Li J., Liu X. (2022). Matrix Vesicles as a Therapeutic Target for Vascular Calcification. Front. Cell Dev. Biol..

[137] Ammirati A.L., Moysés R.M.A., Canziani M.E. (2008). Vascular Calcification in Peritoneal Dialysis Patients. Perit. Dial. Int. J. Int. Soc. Perit. Dial..

[138] Villa-Bellosta R. (2021). Vascular Calcification: Key Roles of Phosphate and Pyrophosphate. Int. J. Mol. Sci..

[139] Kenkre J.S., Bassett J.H.D. (2018). The Bone Remodelling Cycle. Ann. Clin. Biochem..

[140] Goettsch C., Iwata H., Aikawa E. (2014). Parathyroid Hormone. Critical Bridge Between Bone Metabolism and Cardiovascular Disease. Arterioscler. Thromb. Vasc. Biol..

[141] Villa-Bellosta R., Sorribas V. (2009). Phosphonoformic Acid Prevents Vascular Smooth Muscle Cell Calcification by Inhibiting Calcium-Phosphate Deposition. Arterioscler. Thromb. Vasc. Biol..

[142] Chen J.J., Zhang J., Cai Y., Zhou Y.B., Wen G.B., Tang C.S., Qi Y.F., Jiang Z.S. (2014). C-Type Natriuretic Peptide Inhibiting Vascular Calcification Might Involve Decreasing Bone Morphogenic Protein 2 and Osteopontin Levels. Mol. Cell. Biochem..

[143] Zhou Y.B., Gao Q., Li P., Han Y., Zhang F., Qi Y.F., Tang C.S., Gao X.Y., Zhu G.Q. (2013). Adrenomedullin Attenuates Vascular Calcification in Fructose-Induced Insulin Resistance Rats. Acta Physiol..

[144] Kang Y.H., Jin J.S., Yi D.W., Son S.M. (2010). Bone Morphogenetic Protein-7 Inhibits Vascular Calcification Induced by High Vitamin D in Mice. Tohoku J. Exp. Med..

[145] Ter Braake A.D., Shanahan C.M., De Baaij J.H.F. (2017). Magnesium Counteracts Vascular Calcification: Passive Interference or Active Modulation?. Arterioscler. Thromb. Vasc. Biol..

[146] Ciceri P., Elli F., Cappelletti L., Tosi D., Savi F., Bulfamante G., Cozzolino M. (2016). Osteonectin (SPARC) Expression in Vascular Calcification: In Vitro and Ex Vivo Studies. Calcif. Tissue Int..

[147] Shioi A., Morioka T., Shoji T., Emoto M. (2020). The Inhibitory Roles of Vitamin k in Progression of Vascular Calcification. Nutrients.

[148] Abedi F., Sadeghi M., Omidkhoda N., Kelesidis T., Ramezani J., Samadi S., Mohammadpour A.H. (2023). HDL-Cholesterol Concentration and Its Association with Coronary Artery Calcification: A Systematic Review and Meta-Analysis. Lipids Health Dis..

[149] Nanao-Hamai M., Son B.K., Hashizume T., Ogawa S., Akishita M. (2016). Protective Effects of Estrogen against Vascular Calcification via Estrogen Receptor α-Dependent Growth Arrest-Specific Gene 6 Transactivation. Biochem. Biophys. Res. Commun..

[150] Zhu Y., Tao S., Zhang D., Xiao J., Wang X., Yuan L., Pan H., Wang D. (2022). Association between Fibrinogen/Albumin Ratio and Severity of Coronary Artery Calcification in Patients with Chronic Kidney Disease: A Retrospective Study. PeerJ.

[151] Cheng Z.Y., Ye T., Ling Q.Y., Wu T., Wu G.Y., Zong G.J. (2018). Parathyroid Hormone Promotes Osteoblastic Differentiation of Endothelial Cells via the Extracellular Signal-Regulated Protein Kinase 1/2 and Nuclear Factor-ΚB Signaling Pathways. Exp. Ther. Med..

[152] Chai S.B., Chen Y., Xin S.X., Yuan N., Liu Y.F., Sun J.B., Meng X.Y., Qi Y.F. (2021). Positive Association of Leptin and Artery Calcification of Lower Extremity in Patients With Type 2 Diabetes Mellitus: A Pilot Study. Front. Endocrinol..

[153] Tanikawa T., Okada Y., Tanikawa R., Tanaka Y. (2009). Advanced Glycation End Products Induce Calcification of Vascular Smooth Muscle Cells through RAGE/P38 MAPK. J. Vasc. Res..

[154] Zhu D., Rashdan N.A., Chapman K.E., Hadoke P.W., MacRae V.E. (2016). A Novel Role for the Mineralocorticoid Receptor in Glucocorticoid Driven Vascular Calcification. Vascul. Pharmacol..

[155] Zhao Y., Sun Z., Li L., Yuan W., Wang Z. (2022). Role of Collagen in Vascular Calcification. J. Cardiovasc. Pharmacol..

[156] Ding H.T., Wang C.G., Zhang T.L., Wang K. (2006). Fibronectin Enhances in Vitro Vascular Calcification by Promoting Osteoblastic Differentiation of Vascular Smooth Muscle Cells via ERK Pathway. J. Cell. Biochem..

[157] Dong Q., Chen Y., Liu W., Liu X., Chen A., Yang X., Li Y., Wang S., Fu M., Ou J.S. (2020). 25-Hydroxycholesterol Promotes Vascular Calcification via Activation of Endoplasmic Reticulum Stress. Eur. J. Pharmacol..

[158] Peng Y.Q., Xiong D., Lin X., Cui R.R., Xu F., Zhong J.Y., Zhu T., Wu F., Mao M.Z., Liao X.B. (2017). Oestrogen Inhibits Arterial Calcification by Promoting Autophagy. Sci. Rep..

[159] Chao C.-T., Lin S.H. (2020). Uremic Vascular Calcification: The Pathogenic Roles and Gastrointestinal Decontamination of Uremic Toxins. Toxins.

[160] Stubbs J.R., Liu S., Tang W., Zhou J., Wang Y., Yao X., Quarles L.D. (2007). Role of Hyperphosphatemia and 1,25-Dihydroxyvitamin D in Vascular Calcification and Mortality in Fibroblastic Growth Factor 23 Null Mice. J. Am. Soc. Nephrol..

[161] Prosdocimo D.A., Wyler S.C., Romani A.M., O’Neill W.C., Dubyak G.R. (2010). Regulation of Vascular Smooth Muscle Cell Calcification by Extracellular Pyrophosphate Homeostasis: Synergistic Modulation by Cyclic AMP and Hyperphosphatemia. Am. J. Physiol. Cell Physiol..

[162] Wang P., Pan Y., Yang C., Zhang L., Zhao Z., Ye K., Li L., Xia S., Lu X., Shi H. (2022). TNFα Activation and TGFβ Blockage Act Synergistically for Smooth Muscle Cell Calcification in Patients with Venous Thrombosis via TGFβ/ERK Pathway. J. Cell. Mol. Med..

[163] Tóth A., Balogh E., Jeney V. (2020). Regulation of Vascular Calcification by Reactive Oxygen Species. Antioxidants.

[164] Liu X., Cao F., Liu S., Mi Y., Liu J. (2018). BMP2/Smad Signaling Pathway Is Involved in the Inhibition Function of Fibroblast Growth Factor 21 on Vascular Calcification. Biochem. Biophys. Res. Commun..

[165] Liu Q.F., Yu L.X., Yin X.Y., Ye J.M., Li S.S. (2021). Correlation Between Soluble Klotho and Vascular Calcification in Chronic Kidney Disease: A Meta-Analysis and Systematic Review. Front. Physiol..

[166] Shobeiri N., Bendeck M.P. (2017). Interleukin-1β Is a Key Biomarker and Mediator of Inflammatory Vascular Calcification. Arterioscler. Thromb. Vasc. Biol..

[167] Henaut L., Massy Z.A. (2018). New Insights into the Key Role of Interleukin 6 in Vascular Calcification of Chronic Kidney Disease. Nephrol. Dial. Transplant..

[168] Kozakova M., Morizzo C., Jamagidze G., Della Latta D., Chiappino S., Chiappino D., Palombo C. (2023). Association between Low-Density Lipoprotein Cholesterol and Vascular Biomarkers in Primary Prevention. Biomedicines.

[169] Henze L.A., Luong T.T.D., Boehme B., Masyout J., Schneider M.P., Brachs S., Lang F., Pieske B., Pasch A., Eckardt K.U. (2019). Impact of C-Reactive Protein on Osteo-/Chondrogenic Transdifferentiation and Calcification of Vascular Smooth Muscle Cells. Aging.

[170] Liu W., Zhang Y., Yu C.M., Ji Q.W., Cai M., Zhao Y.X., Zhou Y.J. (2015). Current Understanding of Coronary Artery Calcification. J. Geriatr. Cardiol..

[171] Lee S.E., Chang H.J., Sung J.M., Park H.B., Heo R., Rizvi A., Lin F.Y., Kumar A., Hadamitzky M., Kim Y.J. (2018). Effects of Statins on Coronary Atherosclerotic Plaques: The PARADIGM Study. JACC Cardiovasc. Imaging.

[172] Ikegami Y., Inoue I., Inoue K., Shinoda Y., Iida S., Goto S., Nakano T., Shimada A., Noda M. (2018). The Annual Rate of Coronary Artery Calcification with Combination Therapy with a PCSK9 Inhibitor and a Statin Is Lower than That with Statin Monotherapy. npj Aging Mech. Dis..

[173] Généreux P., Madhavan M.V., Mintz G.S., Maehara A., Palmerini T., Lasalle L., Xu K., McAndrew T., Kirtane A., Lansky A.J. (2014). Ischemic Outcomes after Coronary Intervention of Calcified Vessels in Acute Coronary Syndromes: Pooled Analysis from the HORIZONS-AMI (Harmonizing Outcomes with Revascularization and Stents in Acute Myocardial Infarction) and ACUITY (Acute Catheterization. J. Am. Coll. Cardiol..

[174] Li X., Du H., Li J., Chen X. (2023). Intracranial Artery Calcification as an Independent Predictor of Ischemic Stroke: A Systematic Review and a Meta-Analysis. BMC Neurol..

[175] Wang X., Chen X., Chen Z., Zhang M. (2022). Arterial Calcification and Its Association With Stroke: Implication of Risk, Prognosis, Treatment Response, and Prevention. Front. Cell. Neurosci..

[176] Fote G.M., Raefsky S., Mock K., Chaudhari A., Shafie M., Yu W. (2022). Intracranial Arterial Calcifications: Potential Biomarkers of Stroke Risk and Outcome. Front. Neurol..

[177] Palit S., Kendrick J. (2014). Vascular Calcification in Chronic Kidney Disease: Role of Disordered Mineral Metabolism. Curr. Pharm. Des..

[178] Dube P., DeRiso A., Patel M., Battepati D., Khatib-Shahidi B., Sharma H., Gupta R., Malhotra D., Dworkin L., Haller S. (2021). Vascular Calcification in Chronic Kidney Disease: Diversity in the Vesselwall. Biomedicines.

[179] Roumeliotis S., Dounousi E., Salmas M., Eleftheriadis T., Liakopoulos V. (2020). Vascular Calcification in Chronic Kidney Disease: The Role of Vitamin K- Dependent Matrix Gla Protein. Front. Med..

[180] Stabley J.N., Towler D.A. (2017). Arterial Calcification in Diabetes Mellitus: Preclinical Models and Translational Implications. Arterioscler. Thromb. Vasc. Biol..

[181] Harper E., Forde H., Davenport C., Rochfort K.D., Smith D., Cummins P.M. (2016). Vascular Calcification in Type-2 Diabetes and Cardiovascular Disease: Integrative Roles for OPG, RANKL and TRAIL. Vascul. Pharmacol..

[182] Ghosh S., Luo D., He W., Chen J., Su X., Huang H. (2020). Diabetes and Calcification: The Potential Role of Anti-Diabetic Drugs on Vascular Calcification Regression. Pharmacol. Res..

[183] Liabeuf S., Bourron O., Vemeer C., Theuwissen E., Magdeleyns E., Aubert C.E., Brazier M., Mentaverri R., Hartemann A., Massy Z.A. (2015). Erratum to Vascular Calcification in Patients with Type 2 Diabetes: The Involvement of Matrix Gla Protein. Cardiovasc. Diabetol..

[184] Baumgartner H., Hung J., Bermejo J., Chambers J.B., Evangelista A., Griffin B.P., Iung B., Otto C.M., Pellikka P.A., Quiñones M. (2009). Echocardiographic Assessment of Valve Stenosis: EAE/ASE Recommendations for Clinical Practice. Eur. J. Echocardiogr..

[185] Gaisne R., Péré M., Menoyo V., Hourmant M., Larmet-Burgeot D. (2020). Calciphylaxis Epidemiology, Risk Factors, Treatment and Survival among French Chronic Kidney Disease Patients: A Case-Control Study. BMC Nephrol..

[186] Nigwekar S.U., Zhao S., Wenger J., Hymes J.L., Maddux F.W., Thadhani R.I., Chan K.E. (2016). A Nationally Representative Study of Calcific Uremic Arteriolopathy Risk Factors. J. Am. Soc. Nephrol..

[187] Chang J.J. (2019). Calciphylaxis: Diagnosis, Pathogenesis, and Treatment. Adv. Ski. Wound Care.

[188] Olaoye O.A., Koratala A. (2017). Calcific Uremic Arteriolopathy. Oxf. Med. Case Rep..

[189] Roumeliotis S., Roumeliotis A., Dounousi E., Eleftheriadis T., Liakopoulos V. (2020). Biomarkers of Vascular Calcification in Serum. Adv. Clin. Chem..

[190] Clemente A., Traghella I., Mazzone A., Sbrana S., Vassalle C. (2020). Vascular and Valvular Calcification Biomarkers.

[191] Jahnen-Dechent W., Heiss A., Schäfer C., Ketteler M. (2011). Fetuin-A Regulation of Calcified Matrix Metabolism. Circ. Res..

[192] Hamano T., Matsui I., Mikami S., Tomida K., Fujii N., Imai E., Rakugi H., Isaka Y. (2010). Fetuin-Mineral Complex Reflects Extraosseous Calcification Stress in CKD. J. Am. Soc. Nephrol..

[193] Cranenburg E.C.M., Vermeer C., Koos R., Boumans M.L., Hackeng T.M., Bouwman F.G., Kwaijtaal M., Brandenburg V.M., Ketteler M., Schurgers L.J. (2008). The Circulating Inactive Form of Matrix Gla Protein (UcMGP) as a Biomarker for Cardiovascular Calcification. J. Vasc. Res..

[194] Pardali E. (2012). TGFβ Signaling and Cardiovascular Diseases. Int. J. Biol. Sci..

[195] Burgess K.A., Herrick A.L., Watson R.E.B. (2021). Systemic Sclerosis Skin Is a Primed Microenvironment for Soft Tissue Calcification—A Hypothesis. Rheumatology.

[196] Schmelzer C.E.H., Duca L. (2022). Elastic Fibers: Formation, Function, and Fate during Aging and Disease. FEBS J..

[197] Haarhaus M., Brandenburg V., Kalantar-Zadeh K., Stenvinkel P., Magnusson P. (2017). Alkaline Phosphatase: A Novel Treatment Target for Cardiovascular Disease in CKD. Nat. Rev. Nephrol..

[198] Leonard O., Spaak J., Goldsmith D. (2013). Regression of Vascular Calcification in Chronic Kidney Disease—Feasible or Fantasy? A Review of the Clinical Evidence. Br. J. Clin. Pharmacol..

[199] O’Neill W.C., Lomashvili K.A., Malluche H.H., Faugere M.C., Riser B.L. (2011). Treatment with Pyrophosphate Inhibits Uremic Vascular Calcification. Kidney Int..

[200] Jüppner H. (2011). Phosphate and FGF-23. Kidney Int..

[201] Hortells L., Sosa C., Guillén N., Lucea S., Millán Á., Sorribas V. (2017). Identifying Early Pathogenic Events during Vascular Calcification in Uremic Rats. Kidney Int..

[202] Barinda A.J., Ikeda K., Hirata K.I., Emoto N. (2017). Macrophages Highly Express Carbonic Anhydrase 2 and Play a Significant Role in Demineralization of the Ectopic Calcification. Kobe J. Med. Sci..

[203] Adeva-Andany M.M., Fernández-Fernández C., Sánchez-Bello R., Donapetry-García C., Martínez-Rodríguez J. (2015). The Role of Carbonic Anhydrase in the Pathogenesis of Vascular Calcification in Humans. Atherosclerosis.

[204] Silaghi C.N., Ilyés T., Van Ballegooijen A.J., Crăciun A.M. (2020). Calciprotein Particles and Serum Calcification Propensity: Hallmarks of Vascular Calcifications in Patients with Chronic Kidney Disease. J. Clin. Med..

[205] Kutikhin A.G., Feenstra L., Kostyunin A.E., Yuzhalin A.E., Hillebrands J.L., Krenning G. (2021). Calciprotein Particles: Balancing Mineral Homeostasis and Vascular Pathology. Arterioscler. Thromb. Vasc. Biol..

[206] Bundy J.D., Cai X., Mehta R.C., Scialla J.J., de Boer I.H., Hsu C.Y., Go A.S., Dobre M.A., Chen J., Rao P.S. (2019). Serum Calcification Propensity and Clinical Events in CKD. Clin. J. Am. Soc. Nephrol..

[207] Pluquet M., Kamel S., Choukroun G., Liabeuf S., Laville S.M. (2022). Serum Calcification Propensity Represents a Good Biomarker of Vascular Calcification: A Systematic Review. Toxins.

[208] Hyafil F., Messika-Zeitoun D., Burg S., Rouzet F., Benali K., Iung B., Vahanian A., Le Guludec D. (2012). Detection of 18fluoride Sodium Accumulation by Positron Emission Tomography in Calcified Stenotic Aortic Valves. Am. J. Cardiol..

[209] Jenkins W.S.A., Vesey A.T., Shah A.S.V., Pawade T.A., Chin C.W.L., White A.C., Fletcher A., Cartlidge T.R.G., Mitchell A.J., Pringle M.A.H. (2015). Valvular 18F-Fluoride and 18F-Fluorodeoxyglucose Uptake Predict Disease Progression and Clinical Outcome in Patients with Aortic Stenosis. J. Am. Coll. Cardiol..

[210] Ghorbanihaghjo A., Argani H., Golmohamadi Z., Rashtchizadeh N., Abbasi M.M., Bargahi N., Vatankhah A.M., Sanajou D. (2018). Linkage of Fibroblast Growth Factor 23 and Phosphate in Serum: Phosphate and Fibroblast Growth Factor 23 Reduction by Increasing Dose of Sevelamer. J. Bone Metab..

[211] Adema A.Y., De Jong M.A., De Borst M.H., Ter Wee P.M., Vervloet M.G. (2016). Phosphate Binding Therapy to Lower Serum Fibroblast-Growth-Factor-23 Concentrations in Chronic Kidney Disease: Rationale and Study Design of the Sevelamer on FGF23 Trial (SoFT). Nephron.

[212] Giger E.V., Castagner B., Leroux J.C. (2013). Biomedical Applications of Bisphosphonates. J. Control. Release.

[213] Otero J.E., Gottesman G.S., McAlister W.H., Mumm S., Madson K.L., Kiffer-Moreira T., Sheen C., Millán J.L., Ericson K.L., Whyte M.P. (2013). Severe Skeletal Toxicity from Protracted Etidronate Therapy for Generalized Arterial Calcification of Infancy. J. Bone Miner. Res..

[214] Oikonomaki T., Papasotiriou M., Ntrinias T., Kalogeropoulou C., Zabakis P., Kalavrizioti D., Papadakis I., Goumenos D.S., Papachristou E. (2019). The Effect of Vitamin K2 Supplementation on Vascular Calcification in Haemodialysis Patients: A 1-Year Follow-up Randomized Trial. Int. Urol. Nephrol..

[215] de Vriese A.S., Caluwé R., Pyfferoen L., de Bacquer D., de Boeck K., Delanote J., de Surgeloose D., van Hoenacker P., van Vlem B., Verbeke F. (2020). Multicenter Randomized Controlled Trial of Vitamin K Antagonist Replacement by Rivaroxaban with or without Vitamin K2 in Hemodialysis Patients with Atrial Fibrillation: The Valkyrie Study. J. Am. Soc. Nephrol..

[216] Wen W., Portales-Castillo I., Seethapathy R., Durant O., Mengesha B., Krinsky S., Kroshinsky D., Kalim S., Goverman J., Nazarian R.M. (2023). Intravenous Sodium Thiosulphate for Calciphylaxis of Chronic Kidney Disease: A Systematic Review and Meta-Analysis. JAMA Netw. Open.

[217] Djuric P., Dimkovic N., Schlieper G., Djuric Z., Pantelic M., Mitrovic M., Jankovic A., Milanov M., Kuzmanovic Pficer J., Floege J. (2020). Sodium Thiosulphate and Progression of Vascular Calcification in End-Stage Renal Disease Patients: A Double-Blind, Randomized, Placebo-Controlled Study. Nephrol. Dial. Transplant..

[218] Zu Y., Lu X., Song J., Yu L., Li H., Wang S. (2019). Cinacalcet Treatment Significantly Improves All-Cause and Cardiovascular Survival in Dialysis Patients: Results from a Meta-Analysis. Kidney Blood Press. Res..

[219] Torres P.A.U., De Broe M. (2012). Calcium-Sensing Receptor, Calcimimetics, and Cardiovascular Calcifications in Chronic Kidney Disease. Kidney Int..

[220] Ferrer M.D., Ketteler M., Tur F., Tur E., Isern B., Salcedo C., Joubert P.H., Behets G.J., Neven E., D’Haese P.C. (2018). Characterization of SNF472 Pharmacokinetics and Efficacy in Uremic and Non-Uremic Rats Models of Cardiovascular Calcification. PLoS ONE.

[221] Hedayati S.S. (2020). A Novel Treatment for Vascular Calcification in Patients with Dialysis-Dependent Chronic Kidney Disease: Are We There Yet?. Circulation.

[222] Singh A., Tandon S., Tandon C. (2021). An Update on Vascular Calcification and Potential Therapeutics. Mol. Biol. Rep..

[223] Helas S., Goettsch C., Schoppet M., Zeitz U., Hempel U., Morawietz H., Kostenuik P.J., Erben R.G., Hofbauer L.C. (2009). Inhibition of Receptor Activator of NF-κB Ligand by Denosumab Attenuates Vascular Calcium Deposition in Mice. Am. J. Pathol..

